# A Novel Device of Reaching, Grasping, and Retrieving Task for Head-Fixed Mice

**DOI:** 10.3389/fncir.2022.842748

**Published:** 2022-05-12

**Authors:** Satoshi Manita, Koji Ikezoe, Kazuo Kitamura

**Affiliations:** Department of Neurophysiology, University of Yamanashi, Chuo, Japan

**Keywords:** forelimb movements, behavior task, water restriction, agar cube, motor control

## Abstract

Reaching, grasping, and retrieving movements are essential to our daily lives and are common in many mammalian species. To understand the mechanism for controlling this movement at the neural circuit level, it is necessary to observe the activity of individual neurons involved in the movement. For stable electrophysiological or optical recordings of neural activity in a behaving animal, head fixation effectively minimizes motion artifacts. Here, we developed a new device that allows mice to perform reaching, grasping, and retrieving movements during head fixation. In this method, agar cubes were presented as target objects in front of water-restricted mice, and the mice were able to reach, grasp, and retrieve them with their forelimb. The agar cubes were supplied by a custom-made automatic dispenser, which uses a microcontroller to control the two motors to push out the agar cubes. This agar presentation system supplied approximately 20 agar cubes in consecutive trials. We confirmed that each agar cube could be presented to the mouse with an average weight of 55 ± 3 mg and positional accuracy of less than 1 mm. Using this system, we showed that head-fixed mice could perform reaching, grasping, and retrieving tasks after 1 week of training. When the agar cube was placed near the mice, they could grasp it with a high success rate without extensive training. On the other hand, when the agar cube was presented far from the mice, the success rate was initially low and increased with subsequent test sessions. Furthermore, we showed that activity in the primary motor cortex is required for reaching movements in this task. Therefore, our system can be used to study neural circuit mechanisms for the control and learning of reaching, grasping, and retrieving movements under head-fixed conditions.

## Introduction

Rodents frequently perform reaching and grasping movements in their daily lives, such as reaching for a seed on the ground, grabbing it, and bringing it to their mouth to eat. Reaching, grasping, and retrieving objects are achieved through a complex neural mechanism. To grasp an object, the neural system (1) must accurately identify the location of the object using visual, auditory, somatosensory, and olfactory information, (2) needs to generate appropriate motor commands based on the location of the object, and (3) needs to accurately move the muscles necessary to grasp that object. Suppose the object’s position changes from moment to moment while the forelimb is extended. In that case, the neural system must adjust the trajectory of the forelimb according to a control method that can respond to the changes. Reaching movements and related neural activities have been extensively studied in humans and monkeys (Castiello, 2005; Lemon, 2008; Alstermark and Isa, 2012; Churchland et al., 2012; Shenoy et al., 2013; Turella and Lingnau, 2014; Thanawalla et al., 2020). Rodents have also been reported to use their forelimbs to reach, grasp, and retrieve objects (Tsai and Maurer, 1930). Since then, many studies on these movements have long been conducted in rodents (Metz and Whishaw, 2000; Whishaw, 2003; Azim et al., 2014; Wang et al., 2017; Yoshida and Isa, 2018; Lemke et al., 2019; Thanawalla et al., 2020; Manns et al., 2021; Nicola et al., 2021).

To investigate how single neuronal activities are involved in reaching, grasping, and retrieving movements, it is necessary to measure or control their activities during these behaviors. For these purposes, it is useful to utilize a wide variety of transgenic mice expressing calcium sensors or light-activated proteins. Several studies using these transgenic mice have shown correlations and causal relationships between various neural activities and behaviors under head-fixed conditions (Helmchen et al., 2018; Svoboda and Li, 2018). Since head fixation can restrict the behavior of mice compared to freely moving conditions, it is relatively easy to observe the movement of mouse limbs and whiskers and present sensory stimuli. Recently, with head fixation in mice, several studies have shown neuronal activity during reaching, grasping, and retrieving movements (Guo et al., 2015, 2021; Galinanes et al., 2018; Levy et al., 2020; Sauerbrei et al., 2020). In these studies, the mouse was head-fixed and performed a task in which they had to reach, grab, and retrieve a target such as a food pellet or water droplets. This manuscript presents a new method for investigating the reaching, grasping, and retrieving behavior of head-fixed mice. In this method, a single agar cube is automatically supplied in front of a mouse with a restricted water supply, allowing the mouse to reach, grasp, and retrieve it as a target with its forelimb. This system can be used to study the mechanisms of neural circuits for motor control and motor learning under head-fixed conditions.

## Materials and Equipment

### Animals and Surgery

The experiments and procedures were approved by the Animal Experiment Committee of University of Yamanashi. Male and female C57BL/6J mice (>6 weeks old) were purchased from Japan SLC (Hamamatsu, Japan). Female VGAT-ChR2 mice [B6.Cg-Tg(Slc32a1-COP4*H134R/EYFP)8Gfng/J, strain #:014548, The Jackson Laboratory, Bar Harbor, ME, United States] were used for optogenetics experiments. They were kept on a reversed 12-h/12-h light-dark cycle and allowed to eat food freely, and all behavioral experiments were performed in the dark phase. To immobilize the head of a mouse during behavior, we implanted an aluminum head plate [40 mm × 25 mm (the mediolateral: ML and the anteroposterior: AL axis), 1.5 mm thick, with a 15 mm × 10 mm (ML and AL axis) hole in the center or 40 mm × 20 mm, 1.5 mm thick, with a hole of 10 mm diameter in the center] to the head of the mouse. The mouse was anesthetized by inhalation of isoflurane (1.5–3%, 0.2–0.3 L/min), and the body temperature was maintained using a heater. For the surgery we used an artificial cerebrospinal fluid (ACSF) containing: 150 mM NaCl, 2.5 mM KCl, 10 mM HEPES, 2 mM CaCl_2_, and 1 mM MgCl_2_ (pH = 7.4 adjusted with NaOH). Eye ointment (0.3% ofloxacin, TOA Pharmaceuticals, Toyama, Japan) was applied to the eyes of the mice during surgery. The hair on the scalp was removed with a hair removal cream, and a surface anesthetic (Xylocaine Jelly 2%, Aspen Japan, Tokyo, Japan) was applied, and the skull was exposed by incision. The head plate was fixed to the skull with dental cement (Super-bond, SUN Medical, Moriyama, Japan) such that the plate did not cover the eyes of the mice. The exposed skull was covered with dental cement to attach the head plate to the skull and keep the bone from becoming infected with bacteria.

### Behavior System

**FIGURE 1:**
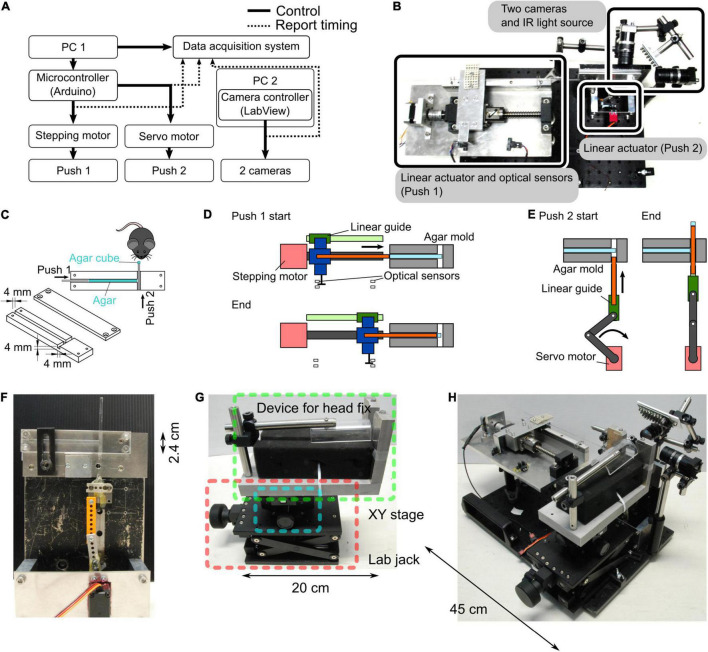
Agar dispenser. **(A)** Configuration of the agar dispenser system. A microcontroller connected to the PC1 regulates the stepping and servo motors, forming two linear actuators. The orthogonal linear actuators push agar in the agar mold twice (Push 1 and 2), which creates an agar cube. We used another PC (PC2) to observe and record mouse behavior with two cameras. Push timings and camera triggers are stored on the PC1 by a data acquisition board. **(B)** Photograph showing the top view of the behavior task system. **(C)** Schematic of the agar mold. Successive pushes by orthogonal plungers (Push 1 → Push 2) create and present the agar cube in front of a mouse. **(D)** A schematic diagram of the molds before and after extrusion of all the agars is shown. Approximately 20 agar cubes are produced from the start to the end of this process. The position of optical sensors determined the start and end positions of the agar extrusion. The main parts are indicated by the following colors; stepping motor: red, linear guide: green, ball screw: dark gray, push 1 plunger: orange, and connecter between linear guide, ball screw, and push 1 plunger: blue. **(E)** Schematic of the push 2. For each agar cube (one trial), the agar extruded by push 1 plunger converted into a cube by push 2. The main parts are color-coded as follows; stepping motor: red, linkage: dark gray, linear guide: green, and push 2 plunger: orange. **(F)** Photograph of a side view of the agar mold and the push 2 mechanism. **(G)** Apparatus for fixing the head of mice. A manual XY stage (blue square) and a laboratory jack (red square) were placed at the bottom of the device that fixes the mouse head (green square) so that the position of the mouse can be adjusted. **(H)** Photograph of the whole system (the agar dispenser system with the head fix apparatus).

We developed a new behavioral experimental system where head-fixed mice could grasp an agar cube using their forelimb (Figure 1). An agar dispenser for making agar cubes consists of an agar mold, two plungers, and a control unit.

#### Agar Mold

The procedure of making an agar cube is as follows: to prepare a 3% agar solution, agar powder (010-15815, FUJIFILM Wako Pure Chemical Corporation, Osaka, Japan) was mixed with deionized water, heated in a microwave oven, and stirred to dissolve. A custom-made mold (outer size (W × H × D): 12 cm × 2.4 cm × 1.2 cm) consists of two acrylic plates. One acrylic plate has a thin channel (4 mm × 4 mm width and depth), and the other covers the channel. These acrylic plates are joined by screws before pouring the agar solution. The prepared agar solution was poured into the mold and hardened at room temperature (Figure 1C).

#### Plungers

The hardened agar was extruded from the agar mold using two orthogonal plungers (push 1 and push 2) to form a cube. The movements of the push 1 and push 2 plungers were controlled by independent self-made linear actuators (Figures 1A–F). The push 1 actuator consists of a stepper motor (SM-42BYG011, Mercury Motor, Shenzhen, China), a linear guide, and a ball screw (Figures 1B,D). To determine the start and end positions of the push 1 plunger, a slot-type photomicrosensor (EE-SX671A, OMRON, Kyoto, Japan) was placed at each location (Figure 1D). The push 2 actuator consists of a servo motor (DS3218, DSSERVO, Dongguan, China), a linear guide, and a slider-crank (Figures 1E,F).

#### Control Unit

A microcontroller (Arduino Uno Rev3, Arduino, Somerville, MA, United States) and a motor drive shield (L293D Motor Drive Shield for Arduino, SainSmart, Las Vegas, NV, United States) attached to the Arduino were used to control the movements of the two motors used in the push 1/2 actuators (Figure 1A). For the movement of the push 1 plunger per trial, the stepper motor rotated 270°, corresponding to a 3.75 mm horizontal displacement calculated by the operating angle per step of the stepping motor: 1.8° (0.025 mm) and travel per rotation of the ball screw: 5 mm. For the movement of the push 2 plunger per trial, the servo motor rotated from 31° to 90°, corresponding to a vertical displacement of 5 mm in 360 ms. The timing of the push 1 and push 2 movements was determined by the experimenter’s keyboard input using a PC connected to the Arduino.

The state parameters of the task, such as the trial number, session start signal, request for trial start trigger, and message to inform the session end, were reported by the PC. Reaching, grasping, and retrieving movements of the mouse were recorded at a 100 Hz frame rate with an infrared LED illumination (an array of 940 nm LED × 56) using two USB3 cameras (BU031, Toshiba TELI, Tokyo, Japan) controlled by custom software written in LabVIEW (National Instruments, Austin, TX, United States).

### Water Restriction

**FIGURE 2:**
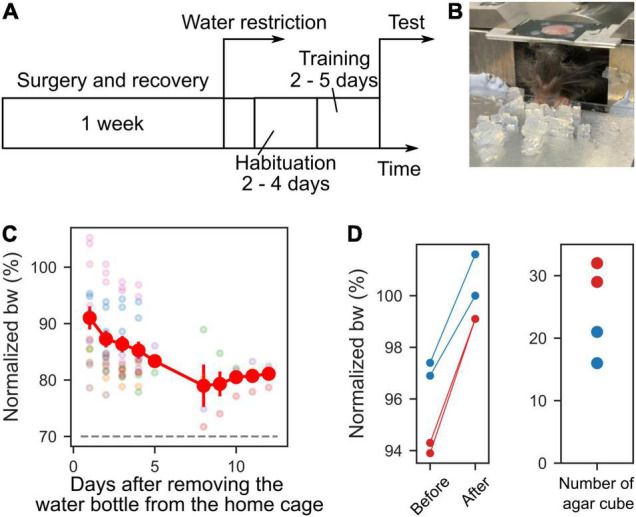
Time course of experiment and body weights. **(A)** Time course of the experiment. One week after the recovery period from the surgery, water restriction was started. Before the test session, habituation and training periods were scheduled. **(B)** Photograph of a mouse during the training. **(C)** Mice body weights (bw) during habituation, training, and test sessions. The body weights were normalized to that on the day water restriction was started. The dashed gray line indicates 70%. Each color represents a different mouse (*n* = 7). **(D)** Left, the normalized weights of four sessions from two mice before they ate the agar cubes and after they could no longer eat them. Right, the number of agar cubes eaten by the mice.

To motivate the mice to grab the agar cubes, we limit the water supply in the following manner. Before starting water restriction, mice were allowed to rest for a week to recover from the surgery to install the head plate (Figure 2A). During rest, the mice were allowed to drink water freely. We removed the water bottle from the cage during the water restriction period, and mice were given 1–2 g of 3% agar cubes instead of water in their home cages, regardless of the consumption of agar cubes during the behavioral sessions. When mice did not train for multiple days, they were given water from a water bottle, and the water supply was restricted the day before training. If mice weighed less than 80% of their weight before starting water restriction, the amount of agar was increased to 3 g per day. If the mice showed signs of dehydration or pain [e.g., ruffled fur or abnormal gait (Guo et al., 2014)], training was stopped, and the water bottle was fed into the home cage until they recovered.

### Habituation and Training Periods

As a preparatory period for behavioral tests, 2–4 days of habituation period were provided to acclimate the mice to the apparatus, followed by 2–5 days of training (Figure 2A). During the habituation period, the experimenter supplied approximately 1 mL of water from a plastic disposable pipette to the mouse under head-fixed conditions in the apparatus for 15–30 min. Some mice drank water from day one, while others needed several days to start drinking. For this reason, the habituation period varied from mouse to mouse and ranged from 2 to 4 days. During the training period, 10–20 agar cubes were administered to the mice simultaneously (Figure 2B). The agar cubes were placed by the experimenter at a distance that was easy for the mice to grasp. Some mice did not reach out and grab and eat the many agars placed in front of mice at the start of training. Even when the mice did not eat the agars, the agar cubes were kept supplied for 1 min, and this was repeated for several days. Then, when mice were able to grab the agar cubes with their forelimbs and eat them (Figure 2B) and gained at least 0.5 g of body weight after 1 day of training, they were moved from the training phase to the test phase. For these reasons, the training period varied from 2 to 5 days, depending on the mouse.

### Reaching, Grasping, and Retrieving Test

After the training period, the reaching, grasping, and retrieving (RGR) test was performed. Agar cubes were placed in front of the mice using the agar dispenser during the test period. One trial was defined as the time when the mouse either acquired or dropped one agar cube, and all trials per day were defined as one session. The position of the mouse and the agar cube was such that the agar cube was directly under the mouse’s jaw, and the agar cube was placed about 2–3 mm to the right of the mouse’s midline so that it could be grasped by the mouse’s right hand. The following steps were performed so that the position of the mouse in relation to the agar dispenser was the same each time. On the first day, we imaged the position of the mouse relative to the agar cube, which was provided by the agar dispenser under head-fixed conditions. We adjusted the position of the mouse in the following sessions based on the position of the eyes and nose in this image. A manual XY linear stage and a laboratory jack built into the head-fixation device were used to adjust the position (Figure 1G). When the agar mold ran out of agar, we replaced it with another mold filled with agar. The mice were kept under head fixation during the exchange of the mold. When the dissolved agar was poured into the mold, some spaces were occasionally created where agar did not exist, presumably due to the contamination with air bubbles. The agar cube could not be supplied to the mouse from this agar-free space. In this case, the trial was considered a catch trial. The trial was excluded from the analysis when an agar cube was small, or two cubes came out simultaneously. The number of trials in each session was to be 40 or 100. If the mouse did not reach for the agar for 1 min, the trial was considered a time-out trial. Two consecutive time-out trials resulted in the termination of the session for that day. If the mouse did not take the agar cube, the experimenter manually removed the agar cube. We conducted at least 13 trials per session during the test period (67.4 ± 32.0 trials/session, 13–103 trials, *n* = 13 mice, 103 sessions). Test sessions were performed under a constant blue light illumination in a light shield box [inner size (W × H × D): 100 cm × 110 cm × 75 cm] unless otherwise stated.

### Agar Cube Measurements

**FIGURE 3:**
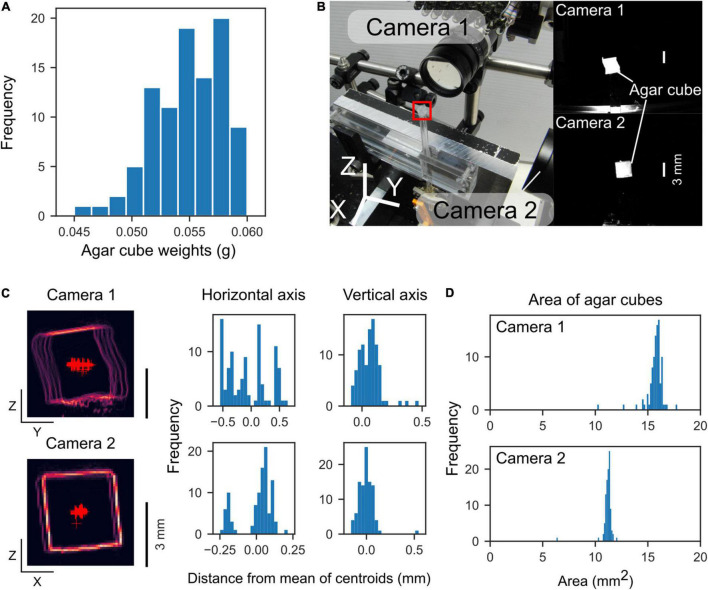
Weight and location of the extruded agar cubes. **(A)** Histogram of agar cube weights (mean: 0.055 g, *n* = 95 cubes, 4 sets). **(B)** Left, Photograph of the measurement configuration. Two orthogonally positioned cameras capture front and side images of the agar cube. The red square indicates the location of an agar cube. Right, Agar cube images by the cameras. **(C)** Left, Outlines of agar cubes captured by the two cameras. The outlines of 99 agar cubes were overlaid. Red “plus” markers indicate centroids of the agar cubes. Right, Histograms of the distance from the mean of centroids along with horizontal and vertical axes. **(D)** Histograms of the area of the agar cubes captured by each camera.

To examine the reproducibility of the agar cubes, we measured the weight, position, and size of the agar cubes produced by the agar dispenser. The operating conditions of the linear actuators for extruding the agar cube were fixed (the stepper motor rotation: 270° per trial, the servo motor angles: 31° and 90° for rest and active states, respectively). The weight of each agar cube was measured individually with an electronic balance. Two cameras captured the front and side images of the agar cubes, from which the area and location of the centroid of the agar cube were measured (Figures 3B–D). At the coordinates in the camera’s field of view, the centroid of 99 agar cubes was measured, and the average coordinates were calculated. The difference between the coordinates of each cube and the average was measured.

### Optogenetic Suppression of the Primary Motor Cortex

To suppress the primary motor cortex (M1) activity, we used mice expressing ChR2 in all GABAergic neurons (VGAT-ChR2 mice) and wild-type mice as control. After completing the training period, the skulls of these mice were thinned with a dental drill. Prior to the light illumination experiment, mineral oil was applied to increase the transparency of the skull. Blue light (470 ± 24 nm) from a fiber-coupled LED photostimulator (Spectra X, Lumencor, Beaverton, OR, United States) was illuminated to the forelimb area of the M1 (0.5 mm anterior and 1.5 mm lateral from the bregma) through the skull. The diameter of the optical fiber core was 1 mm, and the light intensity was 61 mW at the fiber end. The light was delivered as a 40 Hz square wave with a 50% duty cycle for 10 s. The stimulus onset was synchronized with the movement onset of the push 2, which presents the agar cube in front of the mouse. Light illumination trials were randomly chosen with a probability of 20%.

### Analysis

**FIGURE 4:**
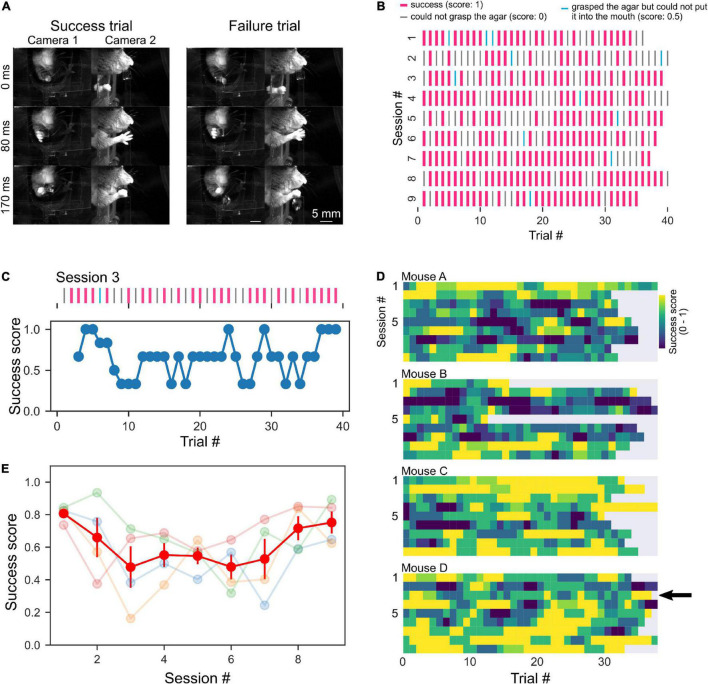
High behavioral performance with short training. **(A)** Sequential photographs of RGR behavior by head-fixed mouse. Examples of success and failure (could not grasp the agar, “type 2 failure”, score: 0) trials of the same mouse are shown. The timestamp represents the time from the beginning of the right forelimb movement of the mouse. **(B)** A representative raster plots of the success and failure trials of a mouse. Pink lines indicate successful trials. Cyan lines indicate trials where the mouse grabbed the cube but dropped it before putting it in its mouth (type 1 failure). Gray lines indicate failure trials where the mouse dropped the cube before grabbing it (type 2 failure). **(C)** A representative example of moving average from raster plot in a session [session 3 shown in panel **(B)**]. **(D)** Color plots of success scores in individual mice. The arrow indicates data in panel **(C)**. **(E)** Mean success scores were plotted across sessions. The light color plots show data from individual mice, and the red plot indicates the average.

**FIGURE 5:**
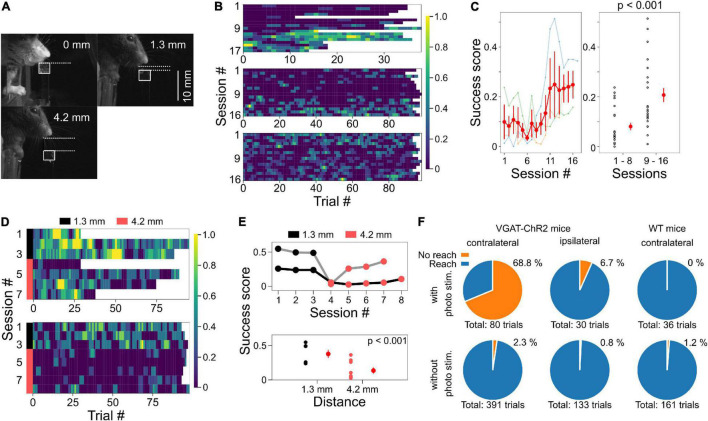
Placing the agar cubes farther away made the task more difficult. **(A)** Examples of different agar cube positions are shown. In each image, the distance from the jaw of the mouse to the agar top is shown. White squares indicate the position of the agar. **(B)** Example of changes in success scores in three mice during test sessions when the agar cube was presented at 1.3 mm from the jaw. **(C)** Left, the relationship between session number and success scores from three mice shown in panel **(B)**. Data plotted in light color represent data from individual mice. Red plots and error bars represent mean and standard error, respectively. Right, Success scores in the second half of the session increased compared to the first half (*p* < 0.001, one-way ANOVA). **(D)** Success scores are shown in color plots when the distance from the mouse jaw to the ager was changed from 1.3 to 4.2 mm. Data obtained from two mice are shown. The black and pink bar lines indicate sessions in which the agars were located at 1.3 and 4.2 mm, respectively. **(E)** Top, from the data in panel **(D)**, the relationship between session number and mean success score are shown. Gray and black lines indicate data from two different mice. Bottom, success scores decreased when the agar cube was placed at 4.2 mm compared to the condition with the agar cube placed at 1.3 mm (*p* < 0.001, one-way ANOVA). **(F)** The reaching movements depend on M1 activity. Left, reaching was suppressed by the photostimulation to M1 on the left (contralateral) side of VGAT-ChR2 mice; with photostim.: 55/80 trials (68.8%), without photostim.: 9/391 trials (2.3%), 2 mice, *p* < 0.001, Chi-square test. Middle, reaching was not suppressed by the photostimulation of M1 on the right (ipsilateral) side; with photostim.: 2/30 trials (6.7%), without photostim.: 1/133 trials (0.8%). Right, reaching was not suppressed by the photostimulation of M1 on the left (contralateral) side of wild-type mice; with photostim.: 0/36 trials (0%), without photostim.: 2/161 trials (1.2%).

The behavior of the mice was manually analyzed by watching the video recorded by the cameras. The results of the trial were classified as success (success score: 1), type 1 failure (grasped the agar but could not put it into the mouth, success score: 0.5), or type 2 failure (unable to grasp the agar, success score: 0), and the mean scores were calculated for each session (Figures 4, 5). To evaluate the time course of success scores during each session, the sequence of success scores was three-point moving averaged which was used as the data for the third window point (Figure 4C). The success score of each session was the mean score of all trials (Figures 4E, 5C,E). To examine whether successful grasping is increased by repetition of sessions, we calculated a parameter called the grab ratio, the number of successful grabs (success and type 1 failure trials) divided by the total number of reach (success, type 1 failure, and type 2 failure trials) in each session. In the optogenetics experiment, we counted the number of trials in which mice extended their forelimb toward the agar cube for 10 s after the agar was presented. Data analysis, statistical testing, and graph creation were performed using custom analysis programs written in Python 3 (SciPy library for statistical testing). All error bars indicate the standard error.

## Results

### Reproducible Agar Cube Production and Presentation

We examined the precision of the reproducibility of the agar cubes produced by the device. The average weight of the agar cube was 0.055 ± 0.003 g (mean ± standard deviation, range: 0.045–0.060 g, *n* = 95 cubes, four agar molds, Figure 3A). The standard deviations of the centroid of the agar cube were 0.36 and 0.088 mm (Y and Z axes) for camera 1, and 0.11 and 0.083 mm (X and Z axes) for camera 2, respectively (*n* = 99 cubes, five agar molds, Figure 3C). The areas of the agar cubes were 16 ± 0.8 mm^2^ for camera 1, and 11 ± 0.54 mm^2^ for camera 2, respectively (Figure 3D). These data showed that the size of agar cubes created by the dispenser was very similar, and each agar cube could be presented with an accuracy of less than 1 mm.

### The Mice Performed the Task Under the Water Restriction

For mice to perform the task, drinking water was restricted, and 3% agar cubes were given as a reward (body weights normalized to those before water restriction: 84.85 ± 5.88%, Figure 2C). After eating the agar cubes in the training or test sessions, the weights of those mice were measured. Even in mice whose body weight was 90% of that before water restriction, the weight gain exceeded 1 g (9 sessions, 2 mice). These results indicate that the mice performed the task with mild water restriction (less than 20% body weight loss) during training and testing periods. We next examined the number of agar cubes that mice could eat. Mice that weighed more than 90% of their pre-restriction weight were given agar cubes until they did not eat anymore. The weight of the mice before and after eating and the number of agar cubes they ate were measured. We found that the mice stopped eating once they reached approximately their pre-restriction weight (2 mice, Figure 2D). This amount corresponded to an average of 24.5 agar cubes (range: 16–32 cubes, *n* = 4 sessions, 2 mice, Figure 2D).

### The Mice Performed the Reaching, Grasping, and Retrieving Task With a High Success Rate in the Post-training Test

During the training period, the mice were given several agar cubes simultaneously and grabbed them (Figure 2B). Even if the mouse could not grab an agar cube once, it was allowed to grab it again as long as the agar cube was within its reach. After 2–5 days of this training, test sessions were conducted. In the test session, the dispenser automatically presented the agar cube, and the mouse was required to grab it and bring it to its mouth. Once the mouse dropped the agar cube, it was unable to grab the same agar cube again. We then examined whether mice performed the task better with the subsequent test session. Unexpectedly, mice performed this task with a high success score even in the early test sessions (success score in session 1: 0.81 ± 0.048, four mice, Figure 4E). Success scores did not change with the number of sessions (one-way ANOVA, *F* = 1.94, *p* = 0.09, Figure 4E) but tended to decrease and then increase again as the number of sessions increased. We also examined whether behavioral performance increased within a single session. However, success scores did not change as the number of trials within a session was increased (one-way ANOVA, *F* = 0.30, *p* = 1.00, *n* = 36 trials in four mice, Figure 4D). These data indicate that trained mice can perform tasks with high performance even when a single agar cube is presented automatically.

We sought to determine why the success rate was very high from the beginning of the testing session and found that the mouse jaw was very close to the agar cube (0 mm away, Figures 4A, 5A). Therefore, we placed the agar cube at a more distant location (1.3 mm away) to make it more difficult for the mice to grasp the agar. As shown in Figures 5B,C, the success scores in the early sessions were low and gradually increased over four sessions by repeated practice (*p* < 0.001, 1–8 sessions: 0.081 ± 0.015, *n* = 24 sessions, three mice, 9–16 sessions: 0.21 ± 0.028, *n* = 24 sessions, three mice, one-way ANOVA, Figure 5C). Moreover, we examined whether placing the ager cube farther away would increase the difficulty of this task. We found that success scores decreased when the agar cube was placed at 4.2 mm compared to those at 1.3 mm (*p* < 0.001, 1.3 mm: 0.38 ± 0.06, *n* = 6 sessions, two mice, 4.2 mm: 0.14 ± 0.04, *n* = 9 sessions, two mice, one-way ANOVA, Figures 5D,E and Supplementary Movies 1, 2). These results indicate that we can change the difficulty of this task by changing the distance between the mice and the agar cubes.

We next examined whether the task could also be used for evaluating the learning of grasping. Mice might not need to learn to grasp the agar cube because the agar cube is softer and presumably easier to grab than the food pellet used in the classical methods. We tested whether agar cube grasping improves with repeated practice by measuring the ratio of successful grasping trials in each session. We found that the grab ratio increased with the number of sessions (*p* < 0.001, one-way ANOVA, 1–8 sessions: 0.10 ± 0.02, *n* = 24 sessions, 9–16 sessions: 0.25 ± 0.03, *n* = 24 sessions, three mice, Supplementary Figure), suggesting that motor skill learning is necessary for grasping the agar cube.

### The Reaching Is Dependent on M1 Activity

To examine whether this task depends on M1 activity, we used transgenic mice expressing ChR2 in GABAergic neurons (VGAT-ChR2 mice). Reaching was suppressed during photostimulation of M1 on the contralateral (left) side of VGAT-ChR2 mice (*p* < 0.001, Chi-square test, with photostim.: 55/80 trials, without photostim.: 9/391 trials, two mice, Figure 5F, left and Supplementary Movie 3). In 37 of the 55 suppressed trials, the forelimb was extended after photostimulation (Supplementary Movie 1). This result is consistent with the results of a previous manuscript (Guo et al., 2015) and is presumably due to rebound activity that activates the motor engram of the trained behavior. On the other hand, reaching movements were not suppressed by photostimulation to the ipsilateral (right side) M1 of the same mice (with photostim.: 2/30 trials, without photostim.: 1/133 trials) or the contralateral M1 of wild-type mice (with photostim.: 0/36 trials, without photostim.: 2/161 trials, Figure 5F, middle and right). These results suggest that the reaching movements and the performance of this task are dependent on the M1 activity.

## Discussion

In this study, we developed a reaching, grasping, and retrieving task device for mice. This device can automatically present agar cubes in front of a head-fixed mouse. This task can be performed in mice with mild water restrictions. Mice could reach, grasp and retrieve the agar cube presented in front at a high success rate without extensive training. The success rate depended on the distance between the mouse and the agar cube. However, with repeated test sessions, the mouse gradually grasped the agar cubes even from a large distance.

### Differences From Previous Methods

Our method allowed mice to perform the task with only drinking water restriction. RGR tasks for rodents generally use food pellets as rewards (Azim et al., 2014; Guo et al., 2015, 2021; Levy et al., 2020; Sauerbrei et al., 2020). To obtain food pellets as rewards during the task, mice must be restricted in their diet, which is a huge burden on rodent health (Heiderstadt et al., 2000; Toth and Gardiner, 2000; Tucci et al., 2006). On the other hand, the agar cube was given as a reward for this behavioral task. Therefore, mice only had restrictions on drinking water, which is less burdensome (Schwarz et al., 2010; Guo et al., 2014; Galinanes et al., 2018), and are more motivated to perform a task for reward (Goltstein et al., 2018). Recently, it has been shown that mice can perform RGR tasks by restricting drinking water (Galinanes et al., 2018). Consistently, we also found that mice can be sufficiently motivated to perform the task by mild restriction of water supply, that is, 1–2 g of agar per day in addition to the agar cubes ingested during the task (∼1 g), instead of total water deprivation. Indeed, mice performed up to ∼100 trials/session without substantial weight loss (∼85% of body weight before training). The number of trials per session showed variability in three out of nine mice tested (Figure 4D Mouse B, Figure 5B top, Figure 5D upper). On the contrary, the number of trials was stable in the other six mice. Therefore, variation in the number of trials is different from mouse to mouse. The reason for this variability is still unclear, and it should be investigated in future studies.

It was reported that using the acetic acid solution as drinking water can maintain mice’s motivation to perform the task while minimizing the burden on their health (Urai et al., 2021). Drinking acetic acid as water control would also be effective in our behavioral task. Furthermore, our method has an additional advantage over the water droplet reaching task (Galinanes et al., 2018). It can evaluate grasping function using solid agar cubes instead of liquid water droplets.

The difficulty of the task can be easily changed using this method. This was achieved by changing the position of the agar cube, which was done by simply changing the displacement of the push 2 plunger (Figure 5A), and thus it can be changed during a session. Therefore, it is possible to investigate more complex functions such as error-based adaptation. Under difficult conditions (4.2 mm ager cube distance), success scores increased slightly as the number of sessions increased (Figure 5E). This result implies that mice adapted to the new location for grasping the agar cubes. Furthermore, our system also allows changing the size of the agar. We expect that this will also change the difficulty of the task.

### M1 Contributes to the Reaching Movements

When M1 in the left hemisphere of VGAT-ChR2 mice was suppressed by photostimulation, reaching movement in the right forelimb was inhibited (Figure 5F and Supplementary Movie 3). This is because GABAergic neurons activated by photostimulation suppressed the surrounding excitatory neurons in M1, and their activity was required to initiate the reaching movements (Zhao et al., 2011). M1 was stimulated for 10 s immediately before the agar cube was presented in front of the mouse. Inhibiting M1 during reaching or grasping may stop the movement (Guo et al., 2015). Changing the intensity and timing of photostimulation to M1 makes it possible to test how the M1 activity affects the different phases of forelimb movements. In addition to M1, behavioral bias similar to that observed in lick movements (Li et al., 2016; Svoboda and Li, 2018) may also be found in forelimb reaching movements by briefly suppressing the premotor cortex before the onset of movement. Different timing or location of photostimulation can provide clues to the neural basis of the complex movement.

### What Can We Do With This Device?

The movements of the mouse are filmed by multiple high-speed (100 Hz) cameras. The markerless pose estimation algorithms such as DeepLabCut (Mathis et al., 2018) allow us to estimate how the mice use their forelimb and fingers to reach and grasp the agar cube and how they change during the learning of the task. Since these movements can be performed under head fixation, simultaneous recording and manipulation of neural activity using a combination of calcium imaging, electrophysiological recordings, and optogenetic perturbation becomes possible. Furthermore, since head fixation suppresses brain vibration, it is effective for measurements that require spatial resolution at the sub-cellular level, such as dendrite and spine imaging (Schwarz et al., 2010; Guo et al., 2014; Burgess et al., 2017; Bjerre and Palmer, 2020; Wagner et al., 2020). These future experiments allow us to unveil the correlation and causal relationship between brain activity and motor parameters or motor learning and to understand the neural circuit mechanisms of these movements.

## Data Availability Statement

The raw data supporting the conclusions of this article will be made available by the authors, without undue reservation.

## Ethics Statement

The animal study was reviewed and approved by the Animal Experiment Committee of University of Yamanashi.

## Author Contributions

SM designed and performed the experiments, analyzed the data, and wrote the manuscript. KI designed the experiments and wrote the manuscript. KK designed the project and wrote the manuscript. All authors contributed to the article and approved the submitted version.

## Conflict of Interest

The authors declare that the research was conducted in the absence of any commercial or financial relationships that could be construed as a potential conflict of interest.

## Publisher’s Note

All claims expressed in this article are solely those of the authors and do not necessarily represent those of their affiliated organizations, or those of the publisher, the editors and the reviewers. Any product that may be evaluated in this article, or claim that may be made by its manufacturer, is not guaranteed or endorsed by the publisher.

## Supplementary Material

The four files are figure, and video recordings of the mouse’s behavior in success (movie_1.mp4), failure (movie_2.mp4) trials, and M1 inhibition with the optogenetic method (movie_3.mp4), respectively.

The following materials created in this study will be deposited and shared in a public repository GitHub (https://github.com/Satoshi-Manita/RGR_test).

• Design files for linear actuators, agar mold, head-fixing apparatus, and head plate.
• Codes for Arduino and LabVIEW files for camera and data acquisition.
