# Supplementary material for: A Novel Device of Reaching, Grasping, and Retrieving Task for Head-Fixed Mice
Source: Front Neural Circuits. 2022 May 12;16:842748. doi: 10.3389/fncir.2022.842748 (PMC9133411; doi:10.3389/fncir.2022.842748)
Supplement: Supplementary file 4 [file Image_1.PDF]

Supplementary figure

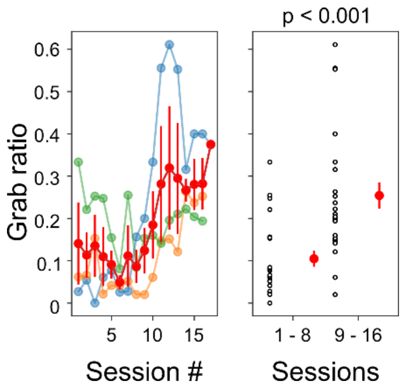


**Supplementary figure: Motor skills are needed to grasp agar cubes.**

Left, relationship between session number and grab ratio from three mice shown in Figure 5B. Data plotted in red represent means and standard errors, while other colors represent individual data. Right, grab ratios in the second half of the session increased compared to the first half. ($p < 0.001$, one-way ANOVA, 1–8 sessions: 0.10 ± 0.02, n = 24 sessions, 9–16 sessions : 0.25 ± 0.03, n = 24 sessions). Red plots and error bars represent mean and standard error, respectively.
